# Application of stochastic fractal search algorithm in novel harmonic blocking filter design for optimizing harmonic mitigation and hosting capacity in electric distribution systems

**DOI:** 10.1371/journal.pone.0320908

**Published:** 2025-05-15

**Authors:** Abdallah Aldosary, Ziad M. Ali, Shady H.E. Abdel Aleem, Ahmed M. Zobaa

**Affiliations:** 1 Department of Computer Engineering, College of Engineering, Prince Sattam bin Abdulaziz University, Ar Riyadh, Saudi Arabia; 2 Department of Electrical Engineering, College of Engineering in Wadi Alddawasir, Prince Sattam Bin Abdulaziz University, Wadi Alddawasir, Saudi Arabia; 3 Department of Electrical Engineering, Institute of Aviation Engineering and Technology, Giza, Egypt; 4 Department of Electrical Power Engineering, Faculty of Engineering, Cairo University, Giza, Egypt; SR University, INDIA

## Abstract

Power networks are being transformed by the incorporation of renewable energy sources (RES), such as photovoltaic systems and wind turbines, which also promote sustainability and lower carbon emissions. However, the widespread use of inverter-based RES threatens power quality and grid stability, with harmonic distortion being a key issue. System performance is compromised by harmonic distortion, elevating the risk of resonance and overheating equipment and increasing power losses. In this study, the Stochastic Fractal Search (SFS) algorithm is used to develop Harmonic Blocking Filters (HBF), an optimized passive power filter for reducing harmonic distortion and minimizing the system active power losses (*P*_*LOSS*_) in electric distribution systems. Two multi-objective optimization algorithms: Multi-Objective Artificial Hummingbird (MOAHA) and Multi-Objective Atomic Orbital Search (MOAOS) efficiently determine the ideal HBF design to maximize the system’s Harmonic-Constrained Hosting Capacity (*HC*_*HC*_) and minimize *P*_*LOSS*_ to support RES while minimizing voltage and current total demand distortion (*THD*_*V*_ and *TDD*_*I*_). Three test systems (TS) are used to validate the HBF’s superiority in mitigating the harmonics, minimizing PLOSS, and maximizing HCHC. In TS1, the SFS-optimized HBF decreases *THD*_*V*_ by 78% and *TDD*_*I*_ by 90% while maintaining *P*_*LOSS*_ almost the same as compared to the previously obtained results in the literature. In TS2, the SFS-optimized HBF decreases *THD*_*V*_ by 16.2%, *TDD*_*I*_ by 99.96%, and *P*_*LOSS*_ by 27.6% compared to the uncompensated case with no filter connected. In TS3, the SFS-optimized HBF decreases *THD*_*V*_ by 45.71%, *TDD*_*I*_ by 99.96%, and *P*_*LOSS*_ by 33.26% compared to the uncompensated case. For the *HC*_*HC*_ enhancement application, MOAOS has proven superior to MOAHA and the MOAOS-optimized HBF increases the system’s *HC*_*HC*_ by 4.18% and in TS3, this value is increased by 16.4% compared to the literature.

## 1. Introduction

The swift incorporation of renewable energy sources (RES) into power networks brings a profound revolution in the worldwide electrical grid. The growing use of inverter-based RES, such as wind turbines and photovoltaic (PV) systems, which are crucial for lowering carbon emissions and promoting sustainable energy development, is driving this shift. However, there are some technological obstacles to overcome before RES can be widely used, especially regarding power quality and system stability. One of the main problems brought on by high RES penetration is harmonic distortion, which can negatively affect power systems’ performance by raising equipment temperatures, increasing power losses, and perhaps creating resonance situations [1–5].

Harmonic distortion is often introduced by nonlinear loads and power electronic devices, such as inverters, which are extensively utilized in modern RES. These gadgets contribute to waveform distortion that spreads throughout the grid by producing voltage and current harmonics in different orders. Harmonic distortion can limit the amount of RES that can be safely integrated without going against harmonic distortion standards set by regulatory bodies like the IEEE and IEC if it is not mitigated. This is because harmonic distortion can lower the network’s effective hosting capacity [6–9].

Many harmonic mitigation strategies, such as active power filters (APFs), hybrid filters, and passive power filters (PPFs), have been developed to solve these harmonic concerns. PPFs are highly appreciated among them due to their ease of use, affordability, and dependability. PPFs are passive filters made of resistors, capacitors, and inductors that are intended to block or attenuate particular harmonic frequencies. In contrast to active filters, which need complicated control systems and external power, PPFs are made of passive components [10–12].

A summary of the literature review on this topic is illustrated in the following paragraphs. Elmi et al. [13] and Ishaya et al. [14] provided a thorough definition and justification for using filters to attenuate harmonic. While active filters are more flexible, they also increase the cost and complexity of the system. In contrast, passive filters are less expensive, easier to use, and capable of adjusting power factors, allowing for good current filtering. The authors stated that they used a single-tuned passive filter to reduce the harmonics because it is widely used for harmonic mitigation. Still, it has several drawbacks that may restrict their ability to lower harmonic distortion in power systems effectively.

Among the main disadvantages is that the single-tuned filter is intended to target a particular harmonic frequency, such as the fifth or seventh harmonic, to achieve limited harmonic suppression. Because of this, they only effectively attenuate at the frequency at which they are tuned and give little suppression for other harmonic orders. Because of this, they are less appropriate for complex harmonic profiles with several harmonic components. Also, it has a resonance risk, where a single-tuned filter is not properly built and tuned, and resonance circumstances may be introduced. This happens when the filter’s resonant frequency coincides with the system’s natural frequency, which causes harmonic currents to be amplified rather than suppressed and may result in equipment damage and overheating. It also has restricted bandwidth, where single-tuned filters are narrow-band; hence, they are unable to accommodate harmonic orders with closely spaced frequencies. Multiple filters are required to cover a greater frequency range since, for example, a filter intended for the 5th harmonic may not sufficiently attenuate the 7th or higher-order harmonics. Also, it suffers less effectiveness at higher harmonic orders; because of the size and expense of the necessary components (such as inductors and capacitors), single-tuned filters are less useful for attenuating high-order harmonics. This makes them less useful in situations where the suppression of high-order harmonics is essential.

The authors in [15] used a passive filter to mitigate the harmonics, they presented detailed modelling of double-tuned filters (DTFs) because they can attenuate many harmonic frequencies at once. Hence, they are commonly used in harmonic mitigation. However, they have several drawbacks that may restrict their usefulness and efficacy in particular situations. One of these drawbacks is that DTFs can introduce resonance sites at other frequencies outside their design range. These resonances can potentially cause harmonic amplification if they are not adequately considered. They can augment some harmonic orders rather than attenuate them if resonance damping is not considered.

Shaikh et al. [16] and Tamaskani et al. [17], in order to reduce harmonics, different kVAR ratings are performed through analysis using a C-type high pass filter. Because of its affordable price and straightforward construction, the C-type filter is frequently utilised for harmonic mitigation. It does, however, have some drawbacks that may affect how well it performs overall in some situations. Among the principal disadvantages is the range of limited harmonic attenuation; because C-type filters are typically made to target particular harmonic frequencies, their effectiveness is limited to a small range of harmonics. Their suitability for systems with a broad range of harmonics or different harmonic orders might be limited, reducing their flexibility in various power network scenarios. It also has problems with resonance; when interacting with the system impedance, C-type filters may cause resonance if not appropriately designed, particularly if the network’s properties alter due to shifting loads or grid reconfigurations. Certain harmonic frequencies may become amplified, which could exacerbate the harmonic distortion rather than lessen it. It affects the power factor, where unwanted changes in the power factor can result from C-type filters, particularly if their size or design are incorrect. This could necessitate more compensatory equipment, making power quality control more difficult overall.

Despite all the efforts in the literature, there is no passive filter capable of simultaneously mitigating voltage and current harmonics and damping harmonic resonance. Hence, this paper introduces the concept of the harmonic blocking filter (HBF). HBFs are a specific kind of PPF that selectively suppress harmonics by combining parallel and series arrangements. In order to efficiently stop undesired harmonic currents from flowing and preventing them from reaching vital components of the power network, the series part of the HBF presents a high impedance path to them. In contrast, the shunt part of the HBF mitigates the harmonic voltages at the point of common coupling (PCC), which is connected parallel to the linear and nonlinear loads and the Distributed Generation (DG). Because of this, HBFs are a desirable alternative for harmonic mitigation in systems with significant RES penetration, where harmonic problems are common because of the way inverters and the grid interact [18–21].

The two separate portions of the HBF used in this study, a series part and a shunt part, each have a particular purpose in harmonic mitigation. The series section combines an inductor and a capacitor, exactly tuned to the fundamental frequency. This arrangement successfully blocks the undesirable harmonic currents while maintaining the intended power flow by allowing the fundamental current component to pass through with the least resistance.

The double resistor damped double-tuned filter (DR-DDTF) is part of the shunt section, which targets voltage harmonics. A certain scheme, named scheme E in [22,23]. It has been chosen due to its efficacy and capability in mitigating voltage harmonics over a broad range of harmonic orders. Due to its double-tuned structure, which enables the simultaneous attenuation of several harmonic frequencies, power quality and harmonic standard compliance are both very efficiently maintained [22,23].

The proposed HBF’s performance and efficiency will be thoroughly validated and evaluated through simulations and analytical studies. By examining its ability to attenuate current and voltage harmonics under varying operating conditions, we aim to understand its effectiveness in harmonic mitigation clearly. This evaluation will include a detailed assessment of the total harmonic distortion (THD) levels, power factor improvement, and the overall stability of the power system when the HBF is integrated.

Following this validation phase, the study will extend its focus to explore the application of the HBF in enhancing the harmonic constraint hosting capacity (HC_HC_) of power networks. The goal is to determine how the deployment of HBFs can maximize the integration potential of inverter-based RES by minimizing harmonic distortions and maintaining compliance with grid standards. This analysis will provide critical insights into the role of harmonic mitigation in facilitating higher RES penetration and ensuring the safe and efficient operation of future power systems. The main contributions of this paper can be summarized as follows:

- The introduction of the HBF to mitigate voltage and current harmonics and damp harmonic resonance simultaneously.
- The utilization of SFS for optimal design of the HBF.
- The validation of the HBF optimal design by SFS using three test systems previously studied in the literature and comparing the results with those obtained.
- The design of the HBF to enhance the *HC*_*HC*_ for two test systems previously investigated in the literature and compare the obtained results with those reported in the literature.

The rest of the paper is organized as follows: The system description is depicted in Section 2. The optimization problem formulation is given in Section 3. The utilized optimization algorithms are described in Section 4. The results and discussions are presented in Section 5. Finally, the conclusions and future work are given in Section 6.

## 2. System description

**Fig 1:**
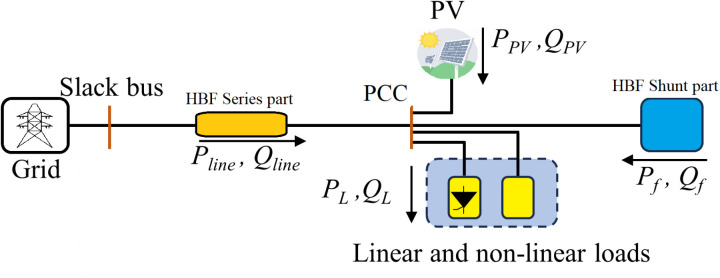
The system under study.

Fig 1 depicts the system under study. The following subsection contains the design and description of the different system components.

### 2.1. Harmonic blocking filter design

HBF is a passive power consisting of two main parts: series and shunt. The shunt part is a damped double-tuned filter (DDTF) for this study. The DDTF has six different schemes (schemes A to E) in the literature [22,24]. The one used in this paper is Scheme E, as it was proven superior to all other schemes [23].

The DTF design equations were illustrated in detail in [15,22,23]. The shunt part of the HBF is a DR-DDTF, and the series part is a series inductor and a capacitor whose design equations are briefly illustrated as follows:


$$\overset{―}{Z_{seriesh}}=\overset{―}{Z_{Lh}}+j{hX}_{Ls}-\frac{jX_{Cs}}{h}\;$$ (1)

$$L_{s}=\frac{1}{\omega_{f}^{2}C_{s}}$$ (2)

$$\overset{―}{Z_{shunth}}=-\frac{jX_{C1}}{h}+(jhX_{L1}//R1)+(-\frac{jX_{C2}}{h}//jhX_{L2}//R_{2})$$ (3)

where *Z*_*seriesh*_ is the equivalent system series impedance, *Z*_*Lh*_ is the harmonic impedance of the line, *h* is the harmonic order, *X*_*Ls*_ is the inductive reactance of the HBF’s series inductor (*L*_*S*_), *X*_*Cs*_ is the capacitive reactance of the HBF’s series capacitor (*C*_*S*_), *ω*_*f*_ is the fundamental angular velocity, *Z*_*shunth*_ is the impedance of the HBF’s shunt part which is the impedance of the DR-DDTF scheme E, *L*_1_, *C*_1_, *L*_2_, *C*_2_, *R*_1_ and *R*_2_ are the DDTF parameters which can be obtained by the following equations:


$$C_{1}=\left(\omega_{f}\left(\frac{\omega_{p}}{\omega_{1}\omega_{2}}\right)-\frac{1}{\omega_{f}}+\frac{\omega_{f}}{\omega_{1}^{2}\omega_{2}^{2}}\left[\frac{\left(\omega_{1}^{2}+\omega_{2}^{2}-\omega_{p}^{2}\right)\omega_{p}^{2}-\omega_{1}^{2}\omega_{2}^{2}}{\omega_{p}^{2}-\omega_{f}^{2}}\right]\right)\times \left(\frac{Q_{f}}{V^{2}}\right)$$ (4)

$$C_{2}=\;C_{1}{\left(\frac{{\omega_{1}}^{2}+{\omega_{2}}^{2}-{\omega_{p}}^{2}}{\omega_{s}^{2}}-1\right)}^{-1}$$ (5)

$$L_{1}=\frac{1}{\omega_{s}^{2}C_{1}}$$ (6)

$$L_{2}=\frac{1}{\omega_{p}^{2}C_{2}}$$ (7)

$$\omega_{s}=\frac{1}{\sqrt{L_{1}C_{1}}}$$ (8)

$$\omega_{p}=\frac{1}{\sqrt{L_{2}C_{2}}}$$ (9)

where Q_f_ is the filter’s reactive power in kVARs, *V* is the PCC’s voltage in kV, ω_s_ and ω_p_ are the tuning angular frequencies of the series and parallel portions of the DDTF, respectively, and ω_1_ and ω_2_ are the two tuning angular frequencies of the DDTF.

### 2.2. Systems under study

Three different systems are studied: TS1, TS2, and TS3. These systems differ in their power rating, voltage level, and harmonic content/signature. The following subsections illustrate the three system details better. The validation of HBF is performed using the three TSs and compared to the obtained results in the literature, and the *HC*_*HC*_ is performed using TS2 and TS3 as they were previously utilized for this purpose in the literature.

#### 2.2.1. First test system (TS1).

The parameters of this system are tabulated in Table 1. The authors used this test system in [15] to validate the undamped DTF design using the slime mould optimization algorithm.

**Table 1:** TS1 parameters.

Parameter	Value
Line voltage (kV)	4.16
Frequency (Hz)	50
Three-phase short circuit capacity (MVA)	150
Three-phase fundamental frequency active power (MW)	5.1
Three phases fundamental frequency reactive power (MVAR)	4.965
Equivalent resistance (Ω)	0.0115
Equivalent reactance (Ω)	0.1154
Resistance of the linear load impedance (Ω)	1.742
Reactance of the linear load impedance (Ω)	1.696
*V*_*S5*_ (V)	76
*V*_*S7*_ (V)	48
*V*_*S11*_ (V)	24
*I*_*L5*_ (A)	99.56
*I*_*L7*_ (A)	44.79
*I*_*L11*_ (A)	19.91
*I*_*L13*_ (A)	9.95

The table shows this system has 13 considered/significant harmonic orders. It uses the grid and the nonlinear load as harmonic sources only. It does not have a DG or PV generator; hence, it will not be utilized for the *HC*_*HC*_ enhancement application.

#### 2.2.2. Second test system (TS2).

This system was previously utilized for the single-resistor DDTF (SR-DDTF) design and Double-Resistor DDTF (DR-DDTF) in [22] and [23], respectively. It was also utilized in the *HC*_*HC*_ enhancement application by the authors in [25]. The parameters of the system are shown in Table 2.

**Table 2:** TS2 parameters.

Parameter	Value
Base voltage (kV)	13.8
Base current (A)	314
Base power (kVA)	7500
Frequency (Hz)	50
Rated load active power (p.u.)	0.92
Rated load reactive power (p.u.)	0.39
Equivalent resistance (Ω)	0.0115
Equivalent reactance (Ω)	0.1154

Based on the system voltage and short-circuit capacity with the corresponding harmonic orders (*h*), Table 3 depicts the values of *IHD*_*I*_ for the non-linear load (*IHD*_*INLL*_) and *IHD*_*V*_ for the grid-side harmonics (*IHD*_*VS*_), also known as background voltage distortion for TS2, along with their maximum limits (*IHD*_*I,MAX*_, *IHD*_*V,MAX*_) implied by IEEE 519 [26]. Additionally, Table 4 depicts the *IHD* values for the PV generator (*IHD*_*IPV*_) utilized in TS2.

**Table 3:** Individual harmonic voltages and currents of non-linear loads, expressed as percentages of their fundamental values, for TS2, and the upper bounds suggested by IEEE 519.

*h*	Non-linear load		Grid-side harmonics	
	*IHD*_*INLL*_ (%)	*IHD*_*I,MAX*_ (%)	*IHD*_*VS*_ (%)	*IHD*_*V,MAX*_ (%)
5	20.00	7.00	3.00	3.00
7	14.30	7.00	2.00	3.00
11	9.10	3.50	2.00	3.00
13	7.70	3.50	1.00	3.00
17	5.90	2.50	1.00	3.00
19	5.30	2.50	1.00	3.00
23	4.30	1.00	1.00	3.00
25	4.00	1.00	0.50	3.00
29	3.40	1.00	0.50	3.00

**Table 4:** The PV generator’s individual harmonic currents as a fraction of the fundamental current in TS2.

*h*	*IHD*_*IPV*_ (%)	*h*	*IHD*_*IPV*_ (%)	*h*	*IHD*_*IPV*_ (%)
1	100	11	0.67	21	0.50
2	1.13	12	0.80	22	0.40
3	3.27	13	0.46	23	0.20
4	0.26	14	1.06	24	0.35
5	3.48	15	0.30	25	1.33
6	0.12	16	0.50	26	0.19
7	1.12	17	1.48	27	0.61
8	0.82	18	0.59	28	1.20
9	0.49	19	1.14	29	0.90
10	0.84	20	0.71	30	0.67

#### 2.2.3. Third test system (TS3).

Table 5 displays the TS3 parameters. Table 6 displays the *IHD*_*INLL*_ values for TS3 and *IHD*_*I,MAX*_ that IEEE 519 suggests based on the system voltage and short-circuit capacity with the corresponding *h*. Table 7 displays the *IHD*_*VS*_ values for TS3 and *IHD*_*VS,MAX*_ that IEEE 519 suggests based on the system voltage and short-circuit capacity related to h. Table 8 displays the *IHD*_*IPV*_ values utilized in TS3.

**Table 5:** TS3 parameters.

Parameter	Value
Base voltage (kV)	11
Base current (A)	525
Base power (kVA)	10000
Frequency (Hz)	50
Rated load active power (p.u.)	0.72
Rated load reactive power (p.u.)	0.38
Thevenin’s resistance (Ω)	0.455
Thevenin’s reactance (Ω)	1.165

**Table 6:** Non-linear load’s individual harmonic currents as a percentage of the fundamental value for TS3.

*h*	*IHD*_*INLL*_ (%)	*h*	*IHD*_*INLL*_ (%)	*h*	*IHD*_*INLL*_ (%)
3	15.00	19	5.30	35	2.43
5	12.00	21	4.74	37	2.21
7	11.00	23	4.32	39	2.06
9	8.05	25	4.01	41	1.88
11	7.15	27	3.79	43	1.64
13	6.42	29	3.40	45	1.47
15	5.87	31	2.86	47	1.35
17	5.44	33	2.62	49	1.26

**Table 7:** The harmonic voltages of the grid expressed as a percentage of the TS3 fundamental value and the upper bounds that IEEE 519 suggests.

*h*	*IHD*_*VS*_ (%)	*IHD*_*V,MAX*_ (%)	*h*	*IHD*_*VS*_ (%)	*IHD*_*V,MAX*_ (%)
h<7	2.00	3.00	25≤h<35	0.50	3.00
7≤h<13	1.25	3.00	35≤h<45	0.25	3.00
13≤≤h<25	0.80	3.00	45≤h<49	0.15	3.00

**Table 8:** The PV generator’s individual harmonic currents as a fraction of the fundamental current in TS3.

*h*	*IHD*_*IPV*_ (%)	*h*	*IHD*_*IPV*_ (%)	*h*	*IHD*_*IPV*_ (%)
2	0.3281	18	0.4344	34	0.0642
3	3.9664	19	1.3261	35	0.2945
4	0.5835	20	0.4288	36	0.0884
5	3.7461	21	1.1262	37	0.2760
6	0.8764	22	0.4066	38	0.0670
7	2.7612	23	0.5980	39	0.2480
8	0.9513	24	0.2670	40	0.0360
9	2.4682	25	0.5322	41	0.2360
10	0.9814	26	0.2150	42	0.0240
11	1.9091	27	0.5030	43	0.2040
12	0.7603	28	0.1970	44	0.0170
13	1.8321	29	0.4850	45	0.1940
14	0.6713	30	0.1690	46	0.0090
15	1.6222	31	0.4240	47	0.1470
16	0.5524	32	0.1120	48	0.0351
17	1.4140	33	0.3970	49	0.1220

### 2.3. System parameters and performance metrics

The source current *I*_*S*_ can be expressed as follows:


$$I_{S}=\sqrt{\sum_{h\geq 1}I_{Sh}^{2}}$$ (10)

where *I*_*Sh*_ is the individual source current harmonics. The PCC voltage *V*_*L*_ can be given as follows:


$$V_{L}=\sqrt{\sum_{h\geq 1}V_{Lh}^{2}}$$ (11)

The current of the DDTF (*I*_*f*_) can be expressed as follows:


$$I_{f}=\sqrt{\sum_{h\geq 1}I_{fh}^{2}}$$ (12)

where *I*_*fh*_ is the DDTF current at *h* and is expressed as:


$$I_{fh}=\frac{\left|V_{Lh}\right|}{\left|Z_{fh}\right|}$$ (13)

The system active power losses *P*_*LOSS*_ can be expressed as:


$$P_{LOSS}=P_{line}+P_{filter}$$ (14)

where *P*_*filter*_ is the active power losses of the filter, which can be calculated as follows, and *P*_*line*_ is the active power losses of the distribution line:


$$P_{line}=\sum_{h\geq 1}\left(I_{sh}^{2}R_{Lh}\right)$$ (15)

The displacement power factor (*DPF*) and real load power factor (*PF*) at *PCC* are calculated as follows:


$$PF=\frac{\sum_{h\geq 1}\left(V_{Lh}\times I_{Sh}\times cos\left(\phi_{h}\right)\right)}{V_{L}\times I_{S}}\times 100$$ (16)

$$DPF=\frac{V_{L1}\times I_{S1}\times cos\left(\varphi_{1}\right)}{V_{L1}\times I_{S1}}\times 100$$ (17)

where $\varphi_{h}$ is the phase between $\overset{―}{V_{Lh}}$ and $\overset{―}{I_{Sh}}$ at *h*, *V*_*L1*_ is the fundamental load voltage, *I*_*S1*_ represents the fundamental source current, and $\varphi_{1}$ is the phase between $\overset{―}{V_{L1}}$ and $\overset{―}{I_{S1}}$. The total harmonic distortion for the PCC voltage (*THD*_*V*_) and the total demand distortion for the source current (*TDD*_*I*_) are expressed, respectively, as follows:


$${THD}_{V}=\frac{\sqrt{\sum_{h\geq 2}V_{Lh}^{2}}}{V_{L1}}\times 100\;$$ (18)

$${TDD}_{I}=\frac{\sqrt{\sum_{h\geq 2}I_{Sh}^{2}}}{I_{LM}}\;\times 100\;$$ (19)

where *I*_*LM*_ represents the fundamental maximum load ampacity. The individual PCC voltage *IHD*_*VL*_ and line current *IHD*_*IL*_ harmonics can be obtained by:


$${IHD}_{VL}=\frac{V_{Lh}}{V_{L1}}\times 100\;$$ (20)

$${IHD}_{IL}=\frac{I_{Sh}}{I_{S1}}\times 100$$ (21)

Finally, *HC*_*HC*_ can be calculated as follows:


$$HC_{HC}=\frac{S_{PV}}{S_{Lrated}}\times 100$$ (22)

where *S*_*PV*_ is the apparent power rating of the PV generator, and *S*_*L*_ rated is the apparent power rating of the load.

## 3. Problem formulation

The problem formulation section is divided into two main subsections: HBF validation stage and *HC*_*HC*_ enhancement using HBF.

### 3.1. HBF design and validation

The objective function *OF*_*1*_ for this purpose can be written as follows:


$$OF_{1}=Min\left(P_{LOSSES}\right)\;(Q_{f},L_{s},L_{1},\;{C_{1},\;L}_{2},{\;C}_{2},\;R_{1},R_{2},{\;h}_{1},\;h_{2},{\;m}_{p})$$ (23)

where *OF*_*1*_ is a function in the filter’s reactive power (*Q*_*f*_), series filter inductance ($L_{s}$), the parallel filter parameters ${(L}_{1},\;{C_{1},\;L}_{2},C_{2},\;R_{1},R_{2})$, the two harmonic tuning orders ${(h}_{1},\;h_{2}$), and the parallel harmonic order (${\;m}_{p})$.

### 3.2. *HC*_*HC*_ Enhancement Using HBF

The *OF*_*2*_ can be expressed as follows:


$$OF_{2}=\left\{ \begin{array}{c} Max\;HC_{HC}(Q_{f}\;,L_{s},{\;L}_{1},\;{C_{1},\;L}_{2},{\;C}_{2},\;R_{1},R_{2},{\;h}_{1},\;h_{2},{\;m}_{p},S_{PV},\emptyset_{PV})\\ Min\;P_{LOSSES}\;({Q_{f}\;,L_{s},{\;L}_{1},\;{C_{1},\;L}_{2},{\;C}_{2},\;R_{1},R_{2},{\;h}_{1},\;h_{2},{\;m}_{p},\;S}_{PV}\emptyset_{PV}) \end{array}\right.\;$$ (24)

where $\varphi_{PV}$ is the PV generator’s power angle. So, determining the optimal values of the *S*_*PV*_ and $\varphi_{PV}$ gives *P*_*PV*_ and *Q*_*PV*_, respectively, the PV generator’s active and reactive powers.

### 3.3. Constraints

The following constraints apply to the earlier objective functions (*Con*):


$${Con}_{1}={V_{rms}}_{min}<{V_{L}}_{rms}<{V_{rms}}_{max}$$ (25)

where ${V_{rms}}_{min}$ and ${V_{rms}}_{max}$ are set to 95% and 105%, respectively.


$${Con}_{2}={I_{L}}_{rms}<{I_{Lrms}}_{max}$$ (26)

where ${I_{Lrms}}_{max}$ is the maximum distribution line ampacity.


$${Con}_{3}=\left\{ \begin{array}{c} {DPF}_{min}<DPF<{DPF}_{max}\\ {PF}_{min}<PF<{PF}_{max} \end{array}\right.\;$$ (27)

where *DPF*_*min*_ and *PF*_*min*_ are set to 0.92, and *DPF*_*max*_ and *PF*_*max*_ are set to 1.


$${Con}_{4}=HC_{HC}\leq 100$$ (28)

$${Con}_{5}=\left\{ \begin{array}{c} {THD}_{V}\leq {{THD}_{V}}_{max}\\ IHD_{VL}\leq {IHD}_{VLmax}\\ {TDD}_{I}\leq {{TDD}_{I}}_{max}\\ IHD_{IL}\leq {IHD}_{ILmax} \end{array}\right.\;$$ (29)

where IEEE 519 implies the upper bounds of the individual and total voltage and current harmonic distortion. *THD*_*Vmax*_ equals 5%, and *TDDI*_*max*_ equals 8%.


$${Con}_{6}=\left\{ \begin{array}{c} L_{1}>0\\ C_{1}>0\\ L_{2}>0\\ C_{2}>0\\ L_{s}>0\\ C_{s}>0 \end{array}\right.\;$$ (30)

## 4. Optimization algorithms

This section briefly summarizes the different utilized optimization algorithms in this work.

### 4.1. Stochastic fractal search (SFS)

Although Fractal Search is a useful technique for issue-solving, it has several disadvantages. The first is that several parameters need to be properly considered, and the second is that particles do not share information. All group members exchange information intending to accelerate convergence to the minimum. Our novel approach tackles this problem by incorporating an updating process phase, as there has not been any information sharing amongst users in fractal search (FS) and the search must be conducted independently. However, there must be a trade-off between accuracy and time consumption because FS is a dynamic algorithm, meaning that the number of agents in the process varies. Stochastic Fractal Search (SFS), an additional variant of Fractal Search, is presented to address the previously noted problems [27].

The two primary functions of the SFS algorithm are distribution and updating processes. To meet the intensification (exploitation) feature, each particle diffuses around its current position in the first phase, much like in Fractal Search. This process increases the likelihood of finding the global minima and prevents one from being trapped in the local minima. The method simulates how a point in the group modifies its position in reaction to the positions of other points in the latter process. In contrast to the diffusing phase of FS, which causes a dramatic increase in the number of participating locations, SFS is considered a static diffusion process. It means that the remaining particles are eliminated, and only the best particle created during the diffusing phase is considered. SFS uses random methodologies as update procedures in addition to its ability to search the issue space rapidly. In other words, the SFS updating process results in exploration characteristics (diversification) in algorithms that use metaheuristics.

Gaussian and Levy flight, two statistical techniques for generating new particles from the diffusion process, are considered. Even though the Levy flight converges faster than the Gaussian Walk in a few generations, preliminary research using the Levy and Gaussian distributions independently demonstrates that the Gaussian Walk has a higher chance of finding global minimum than the Levy flight. SFS’s Diffusion-Limited Aggregation (DLA) generation method only uses the Gaussian distribution for random walks, whereas FS uses the Levy flying distribution. The following equations list the Gaussian walks that are a part of the diffusion process:


$${GW}_{1}=Gaussian\left(\mu_{BP},\sigma \right)+(\varepsilon \times BP-\overset{´}{\varepsilon }\times P_{i})$$ (31)

$${GW}_{2}=Gaussian\left(\mu_{P},\sigma \right)$$ (32)

where the uniformly distributed random integers *ε* and *ε ´* are limited to the interval (0,1). The positions of the group’s best and *i*^*th*^ points are represented by *BP* and *P*_*i*_, respectively. *μ*_*BP*_ and *σ* are the first two Gaussian parameters, and *μ*_*BP*_ is precisely equal to *|BP|*. *μ*_*P*_ and *σ* are the latter parameters, where *μ*_*P*_ equals *|P*_*i*_ *|*. The standard deviation is calculated while taking Gaussian parameters into account in (33), where the function $\frac{log(g)}{g}$ is used to decrease the magnitude of Gaussian jumps as the number of generations increases, promoting a more targeted search as people get closer to the solution.


$$\sigma =\left|\frac{log(g)}{g}\times (P_{i}-BP)\right|\;$$ (33)

Assume that we are currently working on a dimension global optimization issue. Consequently, a D-dimensional vector was used to generate each identified individual believed to be capable of resolving the problem. Throughout the initialization process, each point is initialized at random by establishing minimum and maximum boundaries according to the problem’s requirements. The initialization equation for the *j*^*th*^ point, *P*_*j*_, is handled as follows:


$$P_{j}=LB+\;\varepsilon \;\times (UB-LB)$$ (34)

where LB and UB, respectively, denote the bottom and upper problem-constrained vectors. *ε* is limited to the continuous region [0,1].

To identify the best point (BP) among all points, the fitness function of each point is computed once all points have been initialized. According to the diffusion technique’s exploitation property, every point must be ringed in terms of its current position to use the issue search space. However, due to the exploration attribute, two statistical techniques are considered to enhance space exploration. The first statistical process is applied to each vector index, and the second statistical approach is used for all points. In the first statistical procedure, each point is initially ranked using the value of the fitness function. Next, an equation with a simple uniform distribution is used to express the probability value given to each point *i* in the group:


$${Pa}_{i}=\frac{rank(P_{i})}{N}$$ (35)

where rank (*P*_*i*_) is the rank of point *P*_*i*_ in relation to the other points in the group, and *N* is the total number of points in the group. Equation (35) aims to state that the probability rises as the point’s quality does. Moving points that have not found a workable solution are more likely when this equation is used. Good solutions will, however, be more likely to be transmitted to the next generation. If the condition ${Pa}_{i}<\varepsilon$ is met, the *j*^*th*^ component of *P*_*i*_ for each point *P*_*i*_ in the group is updated in line with (36); otherwise, it remains unchanged.


$$\overset{´}{P_{i}(j)}=P_{r}(j)-\varepsilon \times (P_{t}(j)-P_{i}(j))$$ (36)

where *P*_*i*_’s newly revised position is$\overset{´}{\;P_{i}(j)}$. ε is the random number chosen from the uniform distribution in the continuous space [0,1], and *P*_*r*_ and *P*_*t*_ are randomly chosen points in the group. In connection with the first statistical operation on the points’ constituent parts, the second statistical change seeks to alter a point’s location while considering other points in the group’s position. This quality elevates the bar for investigation and satisfies the diversity criteria. Equation (35) is used to sort all the points obtained from the first statistical technique before starting the second statistical operation. Similar to the last statistical procedure, If the condition ${Pa}_{i}<\varepsilon$ is maintained for a new point $\overset{´}{\;P_{i}(j)}$; the current location of $\overset{´}{P_{i}(j)}$ is updated in line with equations (37) and (38); if not, no update occurs. The following is how equations (37) and (38) are laid out:


$$P_{i}^{\prime \prime }=P_{i}^{\prime }-\hat{\varepsilon }\times \left(P_{t}^{\prime }-BP\right)\;|\;\varepsilon^{\prime }\leq 0.5$$ (37)

$$P_{i}^{\prime \prime }=P_{i}^{\prime }+\hat{\varepsilon }\times \left(P_{t}^{\prime }-P_{r}^{\prime }\right)\;|\;\varepsilon^{\prime }>0.5$$ (38)

where $\hat{\varepsilon }$ are random numbers produced by the Gaussian Normal distribution, and $P_{r}^{\prime }$ and $P_{t}^{\prime }$ are randomly picked points that were obtained by the initial process. If $P_{i}^{\prime }$ its fitness function value is greater than $P_{i}^{\prime \prime }$, $P_{i}^{\prime }$ takes the place of $P_{i}^{\prime \prime }$ as the new point.

### 4.2. Multi-objective atomic orbital search (MOAOS)

Atomic Orbital Search (AOS) was designed to solve single-objective optimization problems and cannot be directly used to tackle multi-objective challenges. As a result, a multi-objective variant of AOS, denoted by MOAOS, is presented for solving multi-criterion optimization problems. Traditionally, heuristic algorithms are used to ﬁnd and store Pareto optimal solutions. However, such solutions are difﬁcult to identify when there are signiﬁcant variations. Hence, a range of alternative approaches to discovering and storing Pareto optimal solutions have been discussed in the literature. AOS was designed to solve single-objective optimization problems and cannot be directly used to tackle multi-objective challenges. As a result, a multi-objective variant of AOS, denoted by MOAOS, is presented in this study to solve multi-criterion optimization problems. Traditionally, heuristic algorithms are used to ﬁnd and store Pareto optimal solutions [27]. However, such solutions are difﬁcult to identify when there are signiﬁcant variations. Hence, a range of alternative approaches to discovering and storing Pareto optimal solutions have been discussed in the literature.

Motivated by the MOPSO method, the MOAOS algorithm incorporates three multi-objective optimization mechanisms to address this challenge:

1 Archive Mechanism:

Derived Pareto optimum solutions can be stored or restored using them as a storage module. Only one controller oversees the archive, and it decides which solutions are added and when they are complete. There is a limit to how many solutions can be stored in the archive. At each iteration, the occupants of the archive are contrasted with the non-dominated solutions created thus far. If at least one archive member controls the new solution, it is not permitted to enter the archive. The new solution might be added to the archive if it overwhelms at least one of the preexisting solutions by leaving out the one that is currently there. The new solution is included in the archive if it does not overshadow the archive solution [1].

2 Grid mechanism:

It is a useful method for improving the archive’s non-dominated solutions. Suppose the archive overflows; one of the solutions can be removed by using the grid technique to reorganize the object space’s partition and identify the most filled area. The extra members should be added to the least crowded segment to boost the final approximate Pareto optimum front variety. As the number of alternative solutions in the hypercube increases, so does the possibility of eliminating a solution. The busiest parts are selected first if the archive is full, and a solution from one of them is randomly deleted to create a way for the new one. A specific instance occurs when a solution is positioned outside of the hypercubes. Every segment has been enlarged in this scenario to accommodate the latest solutions. Consequently, it is also possible to modify the segments of alternate solutions [28].

3 Leader Selection Mechanism:

This technique compares solutions in a multi-objective search space. To find a solution near the global optimum, the search leaders lead the other search candidates to potential regions of the search space. As previously mentioned, the leader selects the least congested regions of the search space and displays the best non-dominated answers, while the archive only includes the best non-dominated solutions. With the following probability, a roulette wheel method is used to choose each hypercube [2]:


$$P_{i}=\frac{C}{N_{i}}$$ (39)

where *C* is a constant number greater than one, and N is the variety of acquired Pareto optimal answers in the *i*^*th*^ section.

Eq. (39) shows that hypercubes with fewer members are more likely to propose new leaders. The likelihood of choosing a hypercube to choose leaders from rises as the number of solutions that can be found in the hypercube decreases. It uses the same mathematical model to find the best answers. It has been shown that search agents must make abrupt position changes at the start of optimization and progressive position changes at the conclusion to obtain optimal solutions using AOS. This tendency guarantees that the algorithm will eventually reach its destination in the search space. Since the MOAOS algorithm considers every element of AOS, every search agent behaves similarly when navigating or taking advantage of the search space. The most important difference is that AOS exclusively stores and enhances the best solutions available, while MOAOS searches around a collection of archive members. MOAOS’s computational complexity is *O*($MN^{2}$), where *M* is the number of objectives, and *N* is the population size. Because MOAOS and MOPSO use archives to hold the best non-dominated solutions, they use more memory than NSGA-II [1], [29–30].

## 5. Results and discussion

This section is divided into two main subsections: The first subsection discusses the filter design and validation using the three test systems (TS1, TS2, and TS3), and the second subsection shows the investigation of the *HC*_*HC*_ enhancement in the last two test systems (TS2 and TS3).

### 5.1. Filter design and validation

The HBF is designed using SFS to minimize *P*_*LOSS*_ in the three different test systems. Testing these systems aims to showcase the filter’s ability to mitigate the harmonics in systems with variant harmonic signatures of the grid voltage and nonlinear load current.

i HBF Design for TS1

The results of designing the HBF filter to minimize *P*_*LOSS*_ in TS1 are shown in Tables 9 and 10. Table 9 compares the different optimization algorithms utilized for HBF design. Those algorithms are SFS, Wild Horse Optimizer (WHO), Grey Wolf Optimizer (GWO), and Genetic Algorithm (GA). The results shown in Table 9 prove that the HBF designed using SFS provides the lowest active power losses; hence, this algorithm will be utilized in further comparisons.

**Table 9:** HBF design results to minimize *P_LOSS_* in TS1 with SFS, WHO, GWO, and GA.

Parameter	SFS	WHO	GWO	GA
*P*_*LOSS*_ (kW)	17.3948	17.7896	18.89	18.6473
Load *PF* (%)	96.8646	96.7247	96.9169	96.9013
*DPF* (%)	96.8674	96.7321	96.9203	96.9043
*THD*_*V*_ (%)	0.80829	0.56195	0.76053	0.77848
*THD*_*I*_ (%)	0.69743	1.1163	0.86751	0.78148
*TDD*_*I*_ (%)	0.4944	0.80016	0.64084	0.57357
*V*_*L*_ (kV)	2.3301148	2.3529131	2.4294823	2.4134674
*I*_*s*_ (A)	708.9043	716.8678	738.7317	733.977

**Table 10:** HBF design results to minimize *P_LOSS_* in TS1.

Parameter	Base Case	Results from using conventional filter designs			Proposed
		MAST	DDM	AM	HBF
*P*_*LOSS*_ (kW)	32.1896	17.3994	17.3995	17.3993	17.3948
Load *PF* (%)	70.9653	98.9021	98.2548	99.487	96.8646
*DPF* (%)	71.6527	99.3515	98.7322	99.9487	96.8674
*THD*_*V*_ (%)	6.0759	3.4236	3.1129	3.5758	0.80829
*THD*_*I*_ (%)	10.2601	7.1631	7.7262	7.1833	0.69743
*TDD*_*I*_ (%)	9.7741	5.1027	5.4872	5.0614	0.4944
*V*_*L*_ (kV)	2.3205	2.4029	2.3805	2.3912	2.3301148
*I*_*s*_ (A)	957.631	714.1831	712.324	706.4221	708.9043

The results of the HBF designed with SFS are compared to those from [3], in which the authors designed an undamped DTF using three different methodologies, namely, Multi-Arm Single-Tuned Filters (MAST), where the DTF is designed as two parallel single-tuned filters, Direct Design Method (DDM), where the DTF is directly designed, and lastly the Analogy Method (AM), where the DTF is designed by placing two-arm single-tuned filter first then comparing the coefficients of their equivalent impedance with the DTF impedance and obtaining the DTF parameters.

The load *PF*, *DPF*, *THD*_*V*_, *THD*_*I*_, and *TDD*_*I*_ values are clearly out of limits in the base case. The HBF almost cancels the voltage and current harmonics, resulting in the lowest *P*_*LOSS*_. The HBF parameters are shown in Table 11.

**Table 11:** HBF design parameters for TS1.

Parameters	HBF
*Q*_*f*_ (MVAR)	1999.8638
*C*_*s*_ (µF)	927.4590
*L*_*s*_ (mH)	10.9246
*C*_*1*_ (mF)	1.1152
*L*_*1*_ (µH)	250.2448
*R*_*1*_ (Ω)	149.0813
*C*_*2*_ (mF)	1.9832
*L*_*2*_ (µH)	52.2862
*R*_*2*_ (Ω)	1017.4947
*h*_*1*_	5.2985
*h*_*2*_	11.2410
*m*_*p*_	9.8850

**Fig 2:**
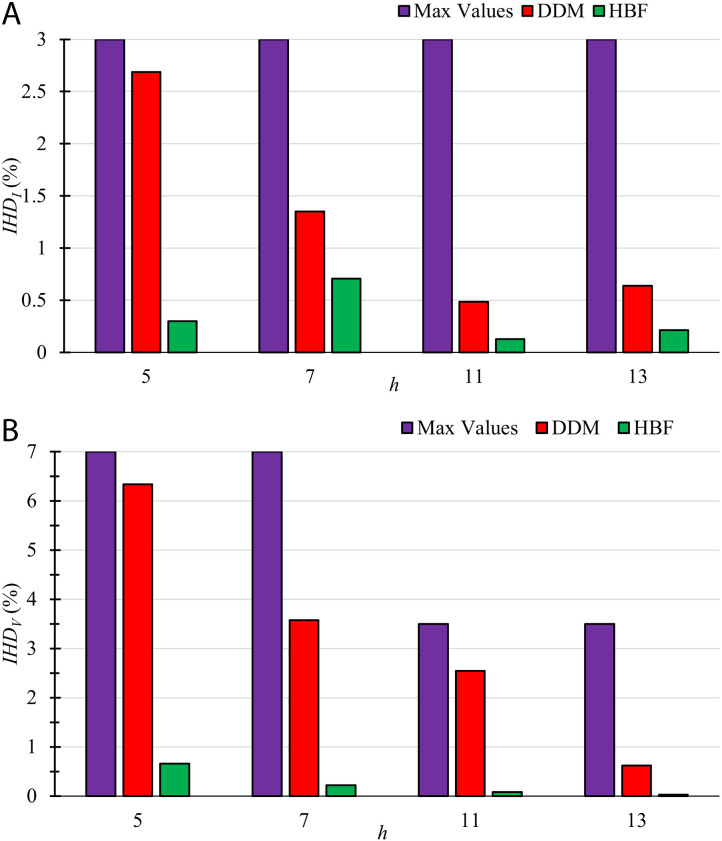
The individual harmonic distortion (a) Currents *IHD*_*I*_ and (b) Voltages *IHD*_*V*_ for TS1. The values of these harmonic currents and voltages are shown using DDM and HBF with the maximum permissible limits by IEEE519-2014.

The individual current *IHD*_*I*_ and voltage *IHD*_*V*_ harmonic distortions are depicted in Fig 2. These values are almost zero in the case of HBF. The values of *IHD*_*I*_ and *IHD*_*V*_ are displayed in the case of DDM, and the maximum individual harmonic voltage and current permissible limits by IEEE 519–2014Click or tap here to enter text. are also shown.

ii HBF Design for TS2

Table 12 displays the results of the HBF design for TS2, along with the base case. The objective is to minimize *P*_*LOSS*_.

**Table 12:** HBF design results minimize *P*_*LOSS*_ in TS2.

Parameter	Base case	HBF
*P*_*LOSS*_ (kW)	282	204.1875
Load *PF* (%)	91.7991	93.3108
*THD*_*V*_ (%)	3.2076	2.688
*TDD*_*I*_ (%)	7.0627	0.0031745
*V*_*L*_ (p.u.)	0.9484	0.98566

The base case *PF*, *THD*_*V*_, *TDD*_*I*_, and *V*_*L*_ are out of limits. HBF minimizes *P*_*LOSS*_ while operating the system within permissible limits. The HBF parameters are depicted in Table 13.

**Table 13:** HBF design parameters for TS2.

Parameters	HBF
*Q*_*f*_ (MVAR)	6.5827
*C*_*s*_ (µF)	13.6042
*L*_*s*_ (mH)	744.7805
*C*_1_ (mF)	850.3504
*L*_1_ (µH)	96.1272
*R*_1_ (Ω)	7562.7414
*C*_2_ (mF)	1951.4027
*L*_2_ (µH)	77.8939
*R*_2_ (Ω)	5060.806
*h*_1_	6.3617
*h*_2_	14.2882
*m*_*p*_	8.1644

**Fig 3:**
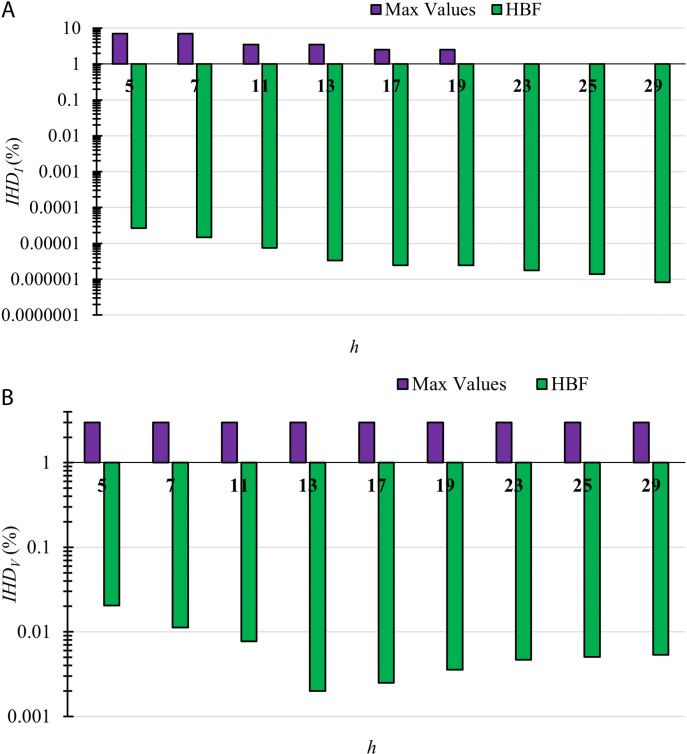
The individual harmonic distortion (a) Currents *IHD*_*I*_ and (b) Voltages *IHD*_*V*_ for TS2. The values of these harmonic currents and voltages are shown when using HBF with the maximum permissible limits by IEEE519-2014. The values are displayed using a logarithmic scale.

The *IHD_I_* and *IHD*_*V*_ for TS2 using the HBF with the maximum permissible limits are displayed in Fig 3, where a logarithmic scale is used to display the values of *IHDI* and *IHD*_*V*_ with the maximum limits as the values are minimal compared to the maximum limits.

The maximum *IHD*_*I*_ limits at harmonic orders 23, 25, and 29 equal 1%, which is why they do not appear on the logarithmic scale. All *IHD*_*I*_ and *IHD*_*V*_ values are less than 1%, which shows the great potential to mitigate both voltage and current harmonics in harmonically distorted power systems.

iii HBF Design for TS3

Table 14 shows the system parameters of TS3 in the base case and using HBF. The load *PF*, *THD*_*V*_, *TDD*_*I*_, and *V*_*L*_ values are beyond limits. All values using HBF are within permissible limits, and the value of *P*_*LOSS*_ is optimized using SFS.

**Table 14:** HBF design results minimize P_LOSS_ in TS3.

Parameter	Base Case	HBF
*P*_*LOSS*_ (kW)	378.61	252.6819
Load *PF* (%)	88.1362	93.6444
*THD*_*V*_ (%)	5.4902	2.9806
*TDD*_*I*_ (%)	6.6486	0.0024413
*V*_*L*_ (p.u.)	0.9469	0.98416

**Fig 4:**
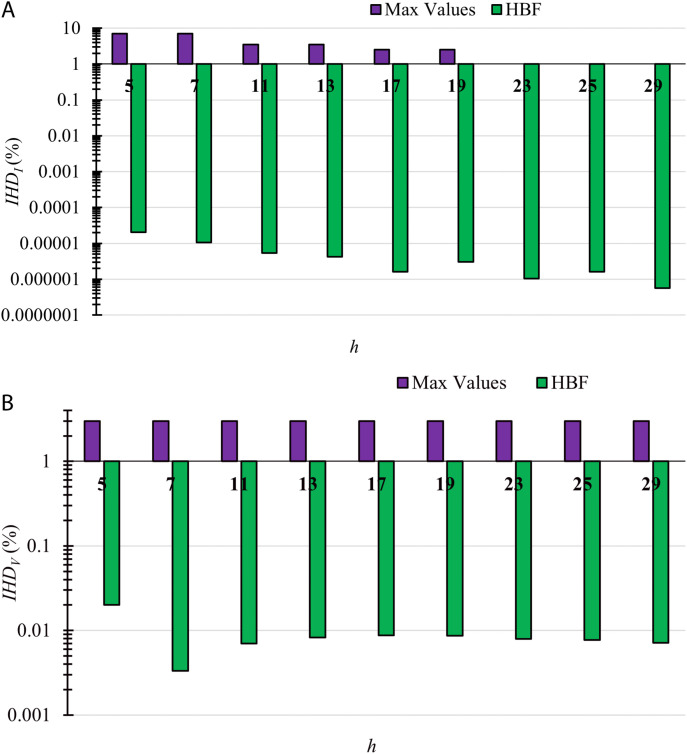
The individual harmonic distortion (a) Currents *IHD*_*I*_ and; (b) Voltages *IHD*_*V*_ for TS3. The values of these harmonic currents and voltages are shown when using HBF with the maximum permissible limits by IEEE519-2014. The values are displayed using a logarithmic scale.

The *IHD*_*I*_ and *IHD*_*V*_ for TS3 using HBF with the maximum permissible limits are displayed in Fig 4, where a logarithmic scale is used to display the values of *IHD*_*I*_ and *IHD*_*V*_ with the maximum limits as the values are minimal compared to the maximum limits. The values of nonlinear load and grid voltage harmonic signatures are zero, starting from harmonic order 30–49, which is why the values appear till the 29th harmonic order only. The main differences between TS2 and TS3 are the system ratings, the individual harmonic nonlinear current and grid voltage, and the inverter-based DG harmonic signatures, which do not appear in these cases as the HBF is designed to minimize *P*_*LOSS*_ in the base case without any DGs presence.

Table 15 shows the HBF design parameters for TS3. The results displayed show that HBF effectively suppresses voltage and current harmonics for different systems with different harmonic signatures. In the next subsection, HBF is used to enhance the *HC*_*HC*_ of DGs in these systems, and its results are compared to those of other filters presented in the literature.

**Table 15:** HBF design parameters for TS3.

Parameters	HBF
*Q*_*f*_ (MVAR)	6.4876
*C*_*s*_ (µF)	11.0244
*L*_*s*_ (mH)	919.0614
*C*_1_ (µF)	898.4122
*L*_1_ (µH)	143.0084
*R*_1_ (Ω)	4352.9975
*C*_2_ (µF)	240.1612
*L*_2_ (µH)	52.7335
*R*_2_ (Ω)	0.31509
*h*_1_	7.5140
*h*_2_	33.4283
*m*_*p*_	28.2850

### 5.2. HC_HC_ enhancement using HBF

In this section, TS2 and TS3 are used, as these systems were previously used in the literature for *HC*_*HC*_ enhancement applications [23]. Table 16 shows the system parameters of TS2 in the base case and using the HBF. Two optimization algorithms, MOAHA and MOAOS, are used to obtain the optimal filter parameters.

**Table 16:** TS2 system parameters for the base case and using HBF for HC_HC_ enhancement.

Parameters	Base case	HBF	
		MOAHA	MOAOS
*PF* (%)	91.7991	94.2687	99.5181
*V*_*L*_ (p.u.)	0.9484	1.0000	1.0004
*P*_*Line*_ (kW)	282	278.0325	795.5389
*P*_*f*_ (kW)	–	8.1786	5.4540
*P*_*LOSS*_ (kW)	282	286.2111	800.9929
*TDD*_*I*_ (%)	7.0627	0.0073	0.0061
*THD*_*V*_ (%)	3.2076	4.8290	4.5355
*HC*_*HC*_ (%)	–	84.3974	85.1584

The *HC*_*HC*_ value is greater than the one reported in [5]. It was 81% using the DDTF scheme E; hence, the utilization of HBF improved the *HC*_*HC*_ value by about 4.16%. The HBF parameters are shown in Table 17. The Pareto front of the MOAOS for TS2 is shown in the S1 file.

**Table 17:** MOAOS-designed HBF parameters for HC_HC_ enhancement in TS2.

Parameters	HBF
*Q*_*f*_ (MVAR)	6.1828
*C*_*s*_ (µF)	10.5937
*L*_*s*_ (mH)	956.4287
*C*_1_ (µF)	776.8819
*L*_1_ (µH)	502.7706
*R*_1_ (kΩ)	72.143
*C*_2_ (µF)	105.5277
*L*_2_ (µH)	155.9367
*R*_2_ (Ω)	23.3902
*h*_1_	4.4324
*h*_2_	28.5129
*m*_*p*_	24.8138

The system parameters of TS3 in the base case and using the HBF are shown in Table 18.

**Table 18:** TS3 system parameters for the base case and using HBF for HC_HC_ enhancement.

Parameters	Base case	HBF	
		MOAHA	MOAOS
*PF* (%)	88.1362	94.1463	92.7654
*V*_*L*_ (p.u.)	0.9469	1.0000	1.0006
*P*_*Line*_ (kW)	378.61	362.7848	85.7490
*P*_*f*_ (kW)	–	0.5626	0.5906
*P*_*LOSS*_ (kW)	378.61	363.3474	86.3396
*TDD*_*I*_ (%)	6.6486	0.0111	0.0052
*THD*_*V*_ (%)	5.4902	3.5120	4.1339
*HC*_*HC*_ (%)	–	91.9677	92.4955

The highest *HC*_*HC*_ that was reported in the literature improved by a passive filter was 76.0796% which was achieved using a MOAHA-designed shunt DR-DDTF Scheme E [23]. This means that the MOAOS-designed HBF improves the *HC*_*HC*_ by 16.4% compared to the highest value in the literature. Table 19 shows the HBF parameters for *HC*_*HC*_ enhancement in TS3. The Pareto front of the MOAOS for TS3 is shown in the S1 file.

**Table 19:** MOAOS-designed HBF parameters for HC_HC_ enhancement in TS3.

Parameters	HBF
*Q*_*f*_ (MVAR)	6.1062
*C*_*s*_ (µF)	28.6246
*L*_*s*_ (mH)	353.9648
*C*_1_ (µF)	1314.8143
*L*_1_ (µH)	301.1429
*R*_1_ (kΩ)	940.5312
*C*_2_ (µF)	680.7859
*L*_2_ (µH)	71.5629
*R*_2_ (kΩ)	939.5703
*h*_1_	4.5007
*h*_2_	16.2088
*m*_*p*_	14.4212

Table 20 shows the effect of different harmonic signatures on the MOAOS-designed HBF. Three cases of the system are shown: there are no grid harmonics while the two other harmonic sources (the nonlinear load and the PV harmonics) are present, there are no nonlinear load harmonics while the other harmonic sources are present, and finally, there are no PV harmonics while the other two harmonic sources are present.

**Table 20:** TS3 System parameters for different cases at which one harmonic source is turned off and the other two are present.

Parameter	No grid harmonics	No nonlinear load harmonics	No PV harmonics	All harmonics included
*PF* (%)	92.7612	92.7765	92.8401	92.7654
*V*_*L*_ (p.u.)	1.0006	1.0004	0.99979	1.0006
*P*_*Line*_ (kW)	85.7476	85.7488	85.7431	85.7490
*P*_*f*_ (kW)	0.5906	0.13943	0.19894	0.5906
*P*_*LOSS*_ (kW)	86.3382	85.8882	85.9421	86.3396
*TDD*_*I*_ (%)	0.004664	0.0051294	0.0023717	0.0052
*THD*_*V*_ (%)	4.1339	3.7505	1.1165	4.1339

In addition, A sensitivity analysis is performed for the HBF parameters in TS3, where the values of all filter capacitors are changed by ± 5%, and the values of all filter inductors are changed by ± 2% [31]. This sensitivity analysis is shown in Table 21.

**Table 21:** TS3 System parameters sensitivity analysis where HBF inductors values are changed by ± 2% and capacitors values by ± 5%.

Parameter	HBF with increased parameters	HBF with decreased parameters	Designed HBF
*PF* (%)	92.7779	92.7487	92.7654
*V*_*L*_ (p.u.)	1.0004	1.0008	1.0006
*P*_*Line*_ (kW)	85.748	85.7502	85.7490
*P*_*f*_ (kW)	0.52791	0.55817	0.5906
*P*_*LOSS*_ (kW)	86.2759	86.3084	86.3396
*TDD*_*I*_ (%)	0.0048143	0.0055973	0.0052
*THD*_*V*_ (%)	3.7775	4.5659	4.1339

From Table 21, one can notice that the system parameters are within the acceptable range by the standards; however, one can also notice a slight improvement in the *THD*_*V*_ and the *P*_*LOSS*_ due to the simultaneous changes in the values of inductors and capacitors.

## 6. Conclusions and future work

This study presented an optimized design of HBFs to reduce harmonic distortion, system *P*_*LOSS*_, and increase *HC*_*HC*_ in electric distribution networks. Because of its exceptional harmonic mitigation capabilities, the HBF—which is made up of a shunt DDTF based on Scheme E and a series inductor-capacitor combination—was chosen. Three test systems (TS1, TS2, and TS3) with different power ratings, voltage levels, and harmonic characteristics were used to validate the filter’s performance and efficiency.

The SFS-optimized HBF successfully decreased *P*_*LOSS*_ while maintaining adherence to allowable power quality constraints, such as load *PF*, *TDD*_*I*_, and *THD*_*V*_ for the three test systems. The MOAOS-optimized HBFs significantly increased *HC*_*HC*_, decreased *P*_*LOSS*_ and effectively suppressed individual harmonic distortions (*IHD*_*I*_ and *IHD*_*V*_) for TS2 and TS3. The outcomes confirm that the suggested HBF architecture is both practicable and resilient in harmonic mitigation, decreasing *P*_*LOSS*_ and enhancing *HC*_*HC*_. The results obtained across three test systems validate the effectiveness of the SFS-optimized HBF in improving power quality, thereby facilitating a more sustainable energy transition. This research underscores the relationship between power quality management and the successful deployment of renewable energy technologies in pursuit of sustainability objectives and grid codes [32].

Practically, the shunt part, which is the DDTF, is well established in industrial applications in research and practical applications. For example, it is used globally in high voltage DC link (HVDC) systems and locally in Cairo Metro. However, the series part of the HBF is still in the prototyping process for MV applications in Egypt and is still under testing, but its practical results will be displayed in future works once it is already implemented. Future studies may also compare the six DDTF schemes for the shunt part of the HBF to improve system performance even more. Other objective functions may be tested rather than *P*_*LOSS*_ and *HC*_*HC*_. More harmonic resonance indices may be investigated to test HBF’s resonance-damping capabilities.

## Supporting information

S1 File The Pareto fronts of MOAOS for HC_HC_ enhancement in TS2 and TS3. (PDF)

S2 File The HBF design code. (DOCX)

## References

[1] El Wejhani S, Elleuch M, Tnani S, Ben Kilani K, Ennine G. Renewable Energy Integration in Power System: Clarification on Stability Indices. 2022 IEEE International Conference on Electrical Sciences and Technologies in Maghreb (CISTEM). 2022:1–6. doi: 10.1109/cistem55808.2022.10044066

[2] B. A. Avdeev, A. V. Vyngra, and V. S. Sobolev. Integration of Renewable Energy Sources into Smart Grids Using Converters Based on Solid- State Transformers. in 2022 5th International Youth Scientific and Technical Conference on Relay Protection and Automation (RPA). 2022, 1–10.

[3] Fadoul FF, Hassan AA, Çağlar R. Assessing the Feasibility of Integrating Renewable Energy: Decision Tree Analysis for Parameter Evaluation and LSTM Forecasting for Solar and Wind Power Generation in a Campus Microgrid. IEEE Access. 2023;11:124690–708. doi: 10.1109/access.2023.3328336

[4] Sindhu M, Madhusudhana J. Challenges in Integration of RES and Control Techniques in Microgrid: A Review. International Journal of Innovative Science and Research Technology (IJISRT). 2024;1716–23.

[5] Vellingiri M, Rawa M, Alghamdi S, Alhussainy AA, Ali ZM, Turky RA, et al. Maximum hosting capacity estimation for renewables in power grids considering energy storage and transmission lines expansion using hybrid sine cosine artificial rabbits algorithm. Ain Shams Engineering Journal. 2023;14(5):102092. doi: 10.1016/j.asej.2022.102092

[6] Almansour M, Drapela J. An Efficient Technique to Reduce Total Harmonics Distortion of Single-Phase Voltage Source Inverter Output Current. 2022 22nd International Scientific Conference on Electric Power Engineering (EPE). 2022:1–6. doi: 10.1109/epe54603.2022.9814155

[7] Almansour M, Drapela J. Complementary Reactive Power Control of Three-Phase AC/DC Voltage Source Converters for Grid Support Applications. 2024 24th International Scientific Conference on Electric Power Engineering (EPE). 2024:1–6. doi: 10.1109/epe61521.2024.10559572

[8] Wei Z, Li J, Wu X, Ye J, Liang Y, Li J. Renewable Energy Hosting Capacity Evaluation of Distribution Network Based on WGAN Scenario Generation. 2023 IEEE International Conference on Power Science and Technology (ICPST). 2023:276–82. doi: 10.1109/icpst56889.2023.10165572

[9] Hassan SJU, Gush T, Kim C-H. Maximum Hosting Capacity Assessment of Distribution Systems With Multitype DERs Using Analytical OPF Method. IEEE Access. 2022;10:100665–74. doi: 10.1109/access.2022.3207488

[10] Mohd Nor KA, Abdullah N. Power quality improvement of three-phase electrical systems using active-passive hybrid harmonic filter. Results in Engineering. 2024;22:102242. doi: 10.1016/j.rineng.2024.102242

[11] Panahi H, Sanaye-Pasand M, Davarpanah M. Three-Terminal Lines Fault Location Using Two Main Terminals Data in the Presence of Renewable Energy Sources. IEEE Trans Smart Grid. 2023;14(3):2085–95. doi: 10.1109/tsg.2022.3216908

[12] Bala Ganesh K, Vijay R, Mathuria P. Ancillary Services from DERs for Transmission and Distribution System Operators. 2022 22nd National Power Systems Conference (NPSC). 2022:482–7. doi: 10.1109/npsc57038.2022.10069115

[13] Elmi Y, Salman D. Simulation Model for Passive Harmonic Filters Using Matlab/Simulink: A Case Study. JPEE. 2023;11(03):1–14. doi: 10.4236/jpee.2023.113001

[14] Ishaya MM, Adegboye OR, Agyekum EB, Elnaggar MF, Alrashed MM, Kamel S. Single-tuned passive filter (STPF) for mitigating harmonics in a 3-phase power system. Sci Rep. 2023;13(1):20754. doi: 10.1038/s41598-023-47614-7 38007548 PMC10676352

[15] Zobaa AM, Abdel Aleem SHE, Youssef HKM. Comparative Analysis of Double-Tuned Harmonic Passive Filter Design Methodologies Using Slime Mould Optimization Algorithm. 1–6.

[16] Shaikh MF, Shaikh AM, Shaikh SA, Khand ZH, Kumar D, Nadeem R. Analysis and Mitigation of Harmonics Using C-Type High Pass Filter and Improvement of Voltage Sag with DVR Including Distributed Generation. 2022 Global Conference on Wireless and Optical Technologies (GCWOT). 2022:1–9. doi: 10.1109/gcwot53057.2022.9772922

[17] Tamaskani R, Khodsuz M, Yazdani-Asrami M. Optimal Design of C-Type Filter in Harmonics Polluted Distribution Systems. Energies. 2022;15(4):1587. doi: 10.3390/en15041587

[18] Cui B, Gao C, Huang L, Ding W, Loh PC. Exploring Non-Convexity Characteristics of Active Trap Filter Based on Local Optimal Control. 2024 IEEE 15th International Symposium on Power Electronics for Distributed Generation Systems (PEDG). 2024, 1–5.

[19] Sou W-K, Martins RP, Lam C-S. Model Predictive Control with Fixed Switching Frequency for Thyristor-Controlled LC-Coupling Hybrid Active Power Filter. 2024 IEEE 33rd International Symposium on Industrial Electronics (ISIE). 2024:1–4. doi: 10.1109/isie54533.2024.10595823

[20] Gono T, Vavra L, Krejci P, Jasinski M, Stacho B. Harmonic Filter Design Methodology. 2024 24th International Scientific Conference on Electric Power Engineering (EPE). 2024, 1–5.

[21] Patra S, Khadem S, Basu M, Komurcugil H, Bayhan S. A Fast Predictive Reference Current Generation Algorithm for Shunt APF in DER Integrated Network. in 2022 3rd International Conference on Smart Grid and Renewable Energy (SGRE). 2022, 1–6.

[22] Zobaa AM, Abdel Aleem SHE, Youssef HKM. Bi-Level damped double-tuned harmonic passive filters design: Multi-criteria decision-making analysis. Ain Shams Engineering Journal. 2023;14(9):102082. doi: 10.1016/j.asej.2022.102082

[23] Alhaider MM, Abdel Aleem SHE, Ali ZM, Zobaa AM. Harmonics management and hosting capacity enhancement: Optimal double-resistor damped double-tuned power filter with artificial hummingbird optimization. PLoS One. 2024;19(5):e0303207. doi: 10.1371/journal.pone.0303207 38728355 PMC11086883

[24] Xiao Y.Algorithm for the parameters of double tuned filter. 154–157.

[25] Ellamsy HT, Ibrahim AM, Ali ZM, Shady AA. Multi-Objective Particle Swarm Optimization for Harmonic-Constrained Hosting Capacity Maximization and Power Loss Minimization in Distorted Distribution Systems. n.d. 1–6.

[26] IEEE. IEEE Recommended Practice and Requirements for Harmonic Control in Electric Power Systems. IEEE Std 519-2014 (Revision of IEEE Std 519-1992). 2014, 1–29.

[27] Cenelec E. Voltage characteristics of electricity supplied by public distribution networks. 2010.

[28] Yang X, Yang Y, Qu D, Chen X, Li Y. Multi-Objective Optimization of Evacuation Route for Heterogeneous Passengers in the Metro Station Considering Node Efficiency. IEEE Trans Intell Transport Syst. 2023;24(11):12448–61. doi: 10.1109/tits.2023.3292912

[29] KarakaŞ MF, LatİFoĞLu F. Metaheuristic FIR Filter Design with Multi-Objective Atomic Orbital Search Algorithm. European Journal of Science and Technology. 2022.

[30] Azizi M, Talatahari S, Giaralis A. Optimization of Engineering Design Problems Using Atomic Orbital Search Algorithm. IEEE Access. 2021;9:102497–519.

[31] Abdel Aleem SHE, Zobaa AF. Optimal C-type filter for harmonics mitigation and resonance damping in industrial distribution systems. Electr Eng. 2016;99(1):107–18. doi: 10.1007/s00202-016-0406-1

[32] Ali ZM, Ćalasan M, Jurado F, Abdel Aleem SHE. Complexities of Power Quality and Harmonic-Induced Overheating in Modern Power Grids Studies: Challenges and Solutions. IEEE Access. 2024;12:151554–97. doi: 10.1109/access.2024.3477729

