# Supplementary material for: Application of stochastic fractal search algorithm in novel harmonic blocking filter design for optimizing harmonic mitigation and hosting capacity in electric distribution systems
Source: PLoS One. 2025 May 15;20(5):e0320908. doi: 10.1371/journal.pone.0320908 (PMC12080870; doi:10.1371/journal.pone.0320908)
Supplement: S1 File — (PDF) [file pone.0320908.s001.pdf]

S1 File. The pareto fronts of MOAOS for HC_{HC} enhancement in TS2 and TS3.

Figure A.1 shows the pareto front of MOAOS for HC_{HC} enhancement in TS2.

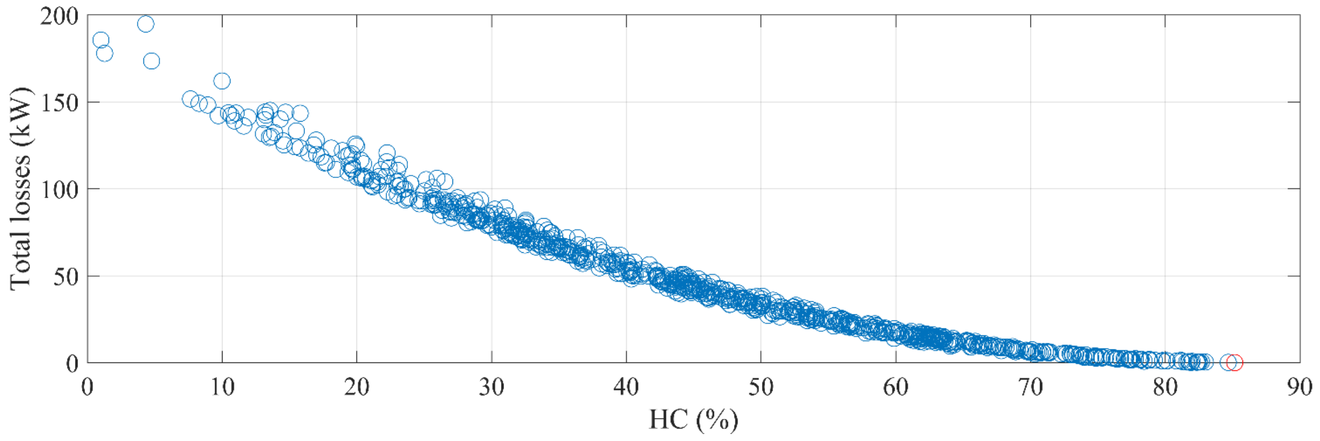

Fig. A.1. The pareto front of MOAOS for HC_{HC} enhancement in TS2.

Figure A.2 shows the pareto front of MOAOS for HC_{HC} enhancement in TS3.

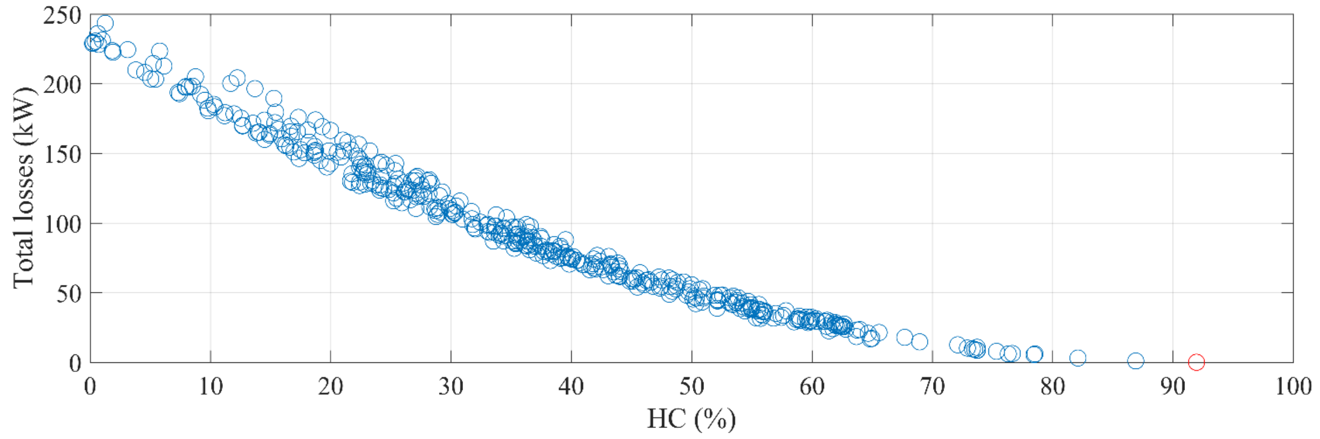

Fig. A.2. The pareto front of MOAOS for HC_{HC} enhancement in TS3.
