# Supplementary material for: Application of stochastic fractal search algorithm in novel harmonic blocking filter design for optimizing harmonic mitigation and hosting capacity in electric distribution systems
Source: PLoS One. 2025 May 15;20(5):e0320908. doi: 10.1371/journal.pone.0320908 (PMC12080870; doi:10.1371/journal.pone.0320908)
Supplement: S2 File — (DOCX) [file pone.0320908.s002.docx]

**S2 File. The HBF design code**

tic

clc;

clear;

global XP

global t_X

XP=1;t_X=1;

MaxIt=50;

nPop=500;

ArchiveSize=500;

FunIndex=6;% ZDT1

[ArchiveFit]=MOAHA(FunIndex,MaxIt,nPop,ArchiveSize);

save('data.mat');

Rank=TOPSIS (XP(:,1:2),[0.5,0.5],[1,0]);

Position_best=find(Rank==max(Rank));

Best=XP(Position_best,:);

XP(:,15)=Rank;

sort_XP=sortrows(XP,15);

pause(0.01);

plot(XP(:,1),XP(:,2),'o','MarkerSize',6,'MarkerSize',10);

hold on

plot(XP(Position_best,1),XP(Position_best,2),'o','MarkerSize',6,'MarkerSize',10,'MarkerEdgeColor','r');

xlabel('HC (%)');

ylabel('Total losses (kW)');

grid on;

pause(0.01);

Time_elapsed=toc

save ('Best_of_all.mat')

SSS=load('Best_of_all.mat');

%% Calculation of filter parameters

basemva = 8.14; accuracy = 0.0001;

maxiter = 100; basekV=13.8;

zbasef=basekV^2/basemva;

% Load_level = 0.7944; % light

Load_level = 1.0; % medium

% Load_level = 1.2997; % heavy

nll_ratio = 0.25; thdv_ratio = 0.972;

RNLL = nll_ratio ; RLL=1 - RNLL; % load non linear part 25% & linear part 75%

Vsub=1.0; % substation voltage

Cable_l=1; % cable length = 2.4*Cable_l (km)

angle_DG=0; % DG'nin harmonik açýsý

angle_NLL=0; % DG'nin harmonik açýsý

%DTF design equations

sb=size(Best);

if sb(1)>1

Q_Shunt=Best(1,3);

h_Shunt1=Best(1,4); %3-4.85

h_Shunt2=Best(1,5); %5-6.4

m_parallel=Best(1,6); %5-7

R1=Best(1,7);

R1=Best(1,8);

SDG=Best(1,9);

Phi_inv = Best(1,10);

else

Q_Shunt=Best(3);

h_Shunt1=Best(4); %3-4.85

h_Shunt2=Best(5); %5-6.4

m_parallel=Best(6); %5-7

R1=Best(7);

R2=Best(8);

SDG=Best(9);

Phi_inv = Best(10);

end

PDG = SDG * cosd(Phi_inv); % active power of DG

QDG = SDG * sind(Phi_inv); % reactive power of DG

busdata=[1 1 Vsub 0.0 0 0 0 0 0 100 0

2 0 1.0 0.0 7.2*Load_level 3.8*Load_level PDG Q_Shunt -10 10 QDG]; % Bus data

linedata=[1 2 0.0337*Cable_l 0.0459*Cable_l 0 1]; % Linedata

Lfybus; % form the bus admittance matrix

Lfnewton; % Power flow solution by Newton-Raphson method @1st frequecny

%% PV harmonic signature

INPV=zeros(1,300);

INPV(10)=177; INPV(20)=1.999; INPV(30)=5.7894; INPV(40)= 0.4644; INPV(50)=6.1646; INPV(60)=0.2201; INPV(70)=1.9876;

INPV(80)=1.4502; INPV(90)=0.8613; INPV(100)= 1.4909; INPV(110)=1.1892; INPV(120)=1.4095; INPV(130)=0.8069; INPV(140)=1.8803;

INPV(150)=0.5389; INPV(160)=0.8896; INPV(170)=2.6213; INPV(180)=1.0392; INPV(190)=2.0138; INPV(200)=1.2602; INPV(210)=0.8827;

INPV(220)=0.7045; INPV(230)=0.3704; INPV(240)= 0.6262; INPV(250)=2.3609; INPV(260)=0.3407; INPV(270)=1.0813; INPV(280)=2.1233;

INPV(290)=1.5887; INPV(300)=1.1931;

INPV=(1/177)*exp(j*angle_DG)*INPV;

%% Nonlinear load harmonic signature

INLL=zeros(1,300);

INLL(10)=100; INLL(50)=20; INLL(70)=14.3; INLL(110)=9.1; INLL(130)=7.7;

INLL(170)=5.9; INLL(190)=5.3; INLL(230)=4.3; INLL(250)=4; INLL(290)=3.4;

INLL=(1/100)*exp(j*angle_NLL)*INLL;

%% Source voltage harmonics

VNLL=zeros(1,300);

VNLL(50)=3; VNLL(70)=2; VNLL(110)=2; VNLL(130)=1;

VNLL(170)=1; VNLL(190)=1; VNLL(230)=1; VNLL(250)=0.5; VNLL(290)=0.5;

VNLL=(1/100)*exp(j*-pi)*VNLL;

harorder=length(VNLL);

h=[1:harorder];

%%-----------------------------------------------------------

%%-----------------------------------------------------------

w1=h_Shunt1*100*pi;

w2=h_Shunt2*100*pi;

wp=m_parallel*100*pi;

ws=(w1*w2)/wp;

w=100*pi;

Qf=basemva*1000*Q_Shunt; %the filter reactive power in KVARs

C1=((((w.^4)/(ws.^2))-((w.^2)*(w1+w2)/(ws.^2))+(wp.^2))*Qf)/((Vm(2)*basekV.^2)*1000*w*((wp.^2)-(w.^2)));

C2=C1*(((((w1.^2)+(w2.^2)-(wp.^2))/(ws.^2))-1).^-1);

L1=((wp/(w1*w2)).^2)*(1/C1);

L2=1/((wp.^2)*C2);

XC1=1/(100*pi*C1);

XL1=100*pi*L1;

XC2=1/(100*pi*C2);

XL2=100*pi*L2;

SUMI=0;SUMVB=0;SUMVS=0;SUMDFCB=0;

for nn=11:300 % harmonic power flow

f=nn/10;

PdpuLL=RLL*Pd(2)/basemva; % pu real poower of load linear part

QdpuLL=RLL*Qd(2)/basemva; % pu reactive poower of load linear part

Qgl = busdata(2,8);

PdpuNLL=RNLL*Pd(2)/basemva; % pu real poower of load non- linear part

QdpuNLL=RNLL*Qd(2)/basemva; % pu reactive poower of load non- linear part

Qgl_inj = busdata(2,11); % added caps or inductor

Qgpu_inj=Qgl_inj /basemva; % per unit value of caps or ind

Qgpu = Qgl/basemva; % PFC capacitor power

Pgpu=Pg(2)/basemva; % pu real poower of gen

VSH(nn)=V(1)*VNLL(nn); % Non linear source voltage

VSHP(nn)=abs(V(1)*VNLL(nn)) * sqrt(2);

IL1=abs((V(1)-V(2))/Z);

ILp1=(V(1)-V(2))/Z;

XCAPF = (Vm(2)^2/Qgpu);

INL(nn)=abs(conj((PdpuNLL+j*QdpuNLL)/Vm(2)))*INLL(nn);

IPV(nn)=(Pgpu/V(2))*INPV(nn); % PV harmonic currents

YL(nn)=PdpuLL/(abs(V(2))^2)-j*QdpuLL/(f*(abs(V(2)^2))); % Load admittance

%Filter impedance

ZL2C2R2(nn)=(((1./R2)+1/(XC2/(f*1i))+(1/(XL2*f*1i))).^-1);

ZL1R1(nn)=((1./R1)+1/(XL1*f*1i))^-1;

ZCC(nn)=ZL2C2R2(nn)+ZL1R1(nn)+(XC1/(f*1i));

YF(nn)=inv(ZCC(nn));

YFD(nn)=1/(R+j*f*X); % Feeder admittance

YFc_inj(nn) = f*j*Qgpu_inj/(Vm(2)^2); % injected reactive power for 11.

YH(nn)=YFD(nn)+YL(nn)+YF(nn)+YFc_inj(nn); % admittance matrice filter admittance will be added YF

IH(nn)=VSH(nn)*YFD(nn)-INL(nn)-IPV(nn); % Current Matris

VBH(nn)=IH(nn)*inv(YH(nn)); % voltage equation [V]=[I]*inv[Y]

ILH(nn)=(VSH(nn)-VBH(nn))*YFD(nn); % Line Current Ih

ILHM(nn)=abs(ILH(nn)); % hth. Load current rms

VBHM(nn)=abs(VBH(nn)); % hth. load bus voltage rms

VSHM(nn)=abs(VSH(nn)); % hth. source bus voltage rms

PFh(nn)=real(VBH(nn)*ILH(nn)); % hth. real power Vh*Ih*cos(phi);

DPf(nn)=abs(real(VBH(nn)*conj(VBH(nn)*YF(nn)))); %hth. filter loss in pu

SUMI=ILHM(nn)^2+SUMI; % sum of hth. line currents

SUMVB=VBHM(nn)^2+SUMVB;

SUMVS=VSHM(nn)^2+SUMVS;

PLD(nn)=VBHM(nn)*ILHM(nn)*cos(angle(VBH(nn))-angle(ILH(nn))); % hth. real power at load bus

QLD(nn)=VBHM(nn)*ILHM(nn)*sin(angle(VBH(nn))-angle(ILH(nn))); % hth. imag power at load bus

PLS(nn)=(ILHM(nn)^2)*real(inv(YFD(nn))); % hth. Line loss

SUMDFCB=(((R*sqrt(f))/R)*((ILHM(nn)/1)^2))+SUMDFCB; % cable derating determine

end

% calculation of parameters

THDV=100*sqrt(SUMVB)/abs(V(2)); % THDV calculation

HDF=((1+SUMDFCB)^(-0.5)); % HDF calculation

VBRMS=sqrt(SUMVB+(abs(V(2))^2)); % pu load bus rms voltage

ILRMS=sqrt(SUMI+(IL1^2)); % pu line current rms value

VSRMS=sqrt(SUMVS+(abs(V(1))^2)); % pu Utility source voltage rms

PLOAD=sum(PLD)+Pd(2)/basemva; % pu load power burada bir deðiþiklik yapýldý. %%%%%%%

QLOAD=sum(QLD)+Q(2);

PF=100*((abs(V(2))*IL1*cos(angle(V(2))-angle(ILp1)))+sum(PLD))/(VBRMS*ILRMS); % Power factor at load bus (0-100)

PFM=1/PF; % to find maximum PF

DPF=100*(abs(V(2))*IL1*cos(angle(V(2))-angle(ILp1)))/(abs(V(2))*IL1); % displacement power factor at load bus

PLINELOSS=(IL1^2)*R+sum(PLS); % pu power loss at line

PFILTERLOSS=sum(DPf); % filter losses

Psystem=PLINELOSS+PFILTERLOSS;

DV=abs(100*(1-VBRMS));

QLOADh=sum(QLD);

QT1=Q(2);

PLev=1/(PDG/8.14); % penetration level estimation

V5H = 100 * VBHM(50);

ihc2=100*(ILHM(20)/1); ihc3=100*(ILHM(30)/1); ihc4=100*(ILHM(40)/1); % Individual limits

ihc5=100*(ILHM(50)/1); ihc16=100*(ILHM(60)/1); ihc7=100*(ILHM(70)/1);

ihc8=100*(ILHM(80)/1); ihc9=100*(ILHM(90)/1); ihc10=100*(ILHM(100)/1);

ihc11=100*(ILHM(110)/1); ihc12=100*(ILHM(120)/1); ihc13=100*(ILHM(130)/1);

ihc14=100*(ILHM(140)/1); ihc15=100*(ILHM(150)/1); ihc16=100*(ILHM(160)/1);

ihc17=100*(ILHM(170)/1); ihc18=100*(ILHM(180)/1); ihc19=100*(ILHM(190)/1);

ihc20=100*(ILHM(200)/1); ihc21=100*(ILHM(210)/1); ihc22=100*(ILHM(220)/1);

ihc23=100*(ILHM(230)/1); ihc24=100*(ILHM(240)/1); ihc25=100*(ILHM(250)/1);

ihc26=100*(ILHM(260)/1); ihc27=100*(ILHM(270)/1); ihc28=100*(ILHM(280)/1);

ihc29=100*(ILHM(290)/1); ihc30=100*(ILHM(300)/1);

TDD=100*sqrt(SUMI)/1; % TDD calculation

YL=PdpuLL/(Vm(2)^2)-j*QdpuLL/(V(2)^2); % Linear Load admittance

YNL = PdpuNLL/(Vm(2)^2)-j*QdpuNLL/(V(2)^2); % nonlinear load admittance

ZLoad = inv(YL); % load impedance

ZNLoad= inv(YNL); % nonlinear load impedance

RLoad=real(ZLoad); RNLoad = real(ZNLoad);

XLoad=imag(ZLoad); XNLoad = imag(ZNLoad);

INLR = abs(conj((PdpuNLL+j*QdpuNLL)/1)); % nonlinear current

INangle=angle(conj((PdpuNLL+j*QdpuNLL)/V(2)));

IR_peak = INLR*sqrt(2);

IR_angle_rad = INangle;

%% Results

%writing results to excel file

A=[VBRMS;HDF;THDV; TDD;PLINELOSS*8.14*1000;PFILTERLOSS*8.14*1000;Psystem*8.14*1000;PF;SDG;cosd(Phi_inv);PDG;(SDG/8.14)*100;Q_Shunt;h_Shunt1;h_Shunt2;m_parallel;C1*1e6;C2*1e6;L1*1e3;L2*1e3;R1;R2];

filename = '..\..\DDTF_results_multi_30_e.xlsx';

sheet='results';

xlRange = 'H14';

HC_old = xlsread(filename,sheet,xlRange);

if ((SDG/8.14)*100>HC_old)

xlRange = 'H3';

xlswrite(filename,A,sheet,xlRange)

end

if isempty(HC_old)

xlRange = 'H3';

xlswrite(filename,A,sheet,xlRange)

end

IHDI=ILHM([20:10:300])'*100;

IHDV=VBHM([20:10:300])'*100;

IHD=[IHDI;IHDV];

if ((SDG/8.14)*100>HC_old)

sheet='IHD';

xlRange = 'H3';

xlswrite(filename,IHD,sheet,xlRange)

end

if isempty(HC_old)

sheet='IHD';

xlRange = 'H3';

xlswrite(filename,IHD,sheet,xlRange)

end
